# Nonhistone Proteins HMGB1 and HMGB2 Differentially Modulate the Response of Human Embryonic Stem Cells and the Progenitor Cells to the Anticancer Drug Etoposide

**DOI:** 10.3390/biom10101450

**Published:** 2020-10-15

**Authors:** Alireza Jian Bagherpoor, Martin Kučírek, Radek Fedr, Soodabeh Abbasi Sani, Michal Štros

**Affiliations:** Institute of Biophysics of the Czech Academy of Sciences, Královopolská 135, 612 00 Brno, Czech Republic; a.bagherpoor@cent.uw.edu.pl (A.J.B.); martin.kucirek@med.muni.cz (M.K.); fedr@ibp.cz (R.F.); sabbasisani@yahoo.com (S.A.S.)

**Keywords:** HMGB1 and HMGB2, human embryonic stem cells, neuroectodermal cells, apoptosis, telomerase, etoposide

## Abstract

HMGB1 and HMGB2 proteins are abundantly expressed in human embryonic stem cells (hESCs) and hESC-derived progenitor cells (neuroectodermal cells, hNECs), though their functional roles in pluripotency and the mechanisms underlying their differentiation in response to the anticancer drug etoposide remain to be elucidated. Here, we show that *HMGB1* and/or *HMGB2* knockdown (KD) by shRNA in hESCs did not affect the cell stemness/pluripotency regardless of etoposide treatments, while in hESC-derived neuroectodermal cells, treatment resulted in differential effects on cell survival and the generation of rosette structures. The objective of this work was to determine whether HMGB1/2 proteins could modulate the sensitivity of hESCs and hESC-derived progenitor cells (hNECs) to etoposide. We observed that *HMGB1* KD knockdown (KD) and, to a lesser extent, *HMGB2* KD enhanced the sensitivity of hESCs to etoposide. Enhanced accumulation of 53BP1 on telomeres was detected by confocal microscopy in both untreated and etoposide-treated *HMGB1* KD hESCs and hNECs, indicating that the loss of *HMGB1* could destabilize telomeres. On the other hand, decreased accumulation of 53BP1 on telomeres in etoposide-treated *HMGB2* KD hESCs (but not in *HMGB2* KD hNECs) suggested that the loss of *HMGB2* promoted the stability of telomeres. Etoposide treatment of hESCs resulted in a significant enhancement of telomerase activity, with the highest increase observed in the *HMGB2* KD cells. Interestingly, no changes in telomerase activity were found in etoposide-treated control hNECs, but *HMGB2* KD (unlike *HMGB1* KD) markedly decreased telomerase activity in these cells. Changes in telomerase activity in the etoposide-treated *HMGB2* KD hESCs or hNECs coincided with the appearance of DNA damage markers and could already be observed before the onset of apoptosis. Collectively, we have demonstrated that HMGB1 or HMGB2 differentially modulate the impact of etoposide treatment on human embryonic stem cells and their progenitor cells, suggesting possible strategies for the enhancement of the efficacy of this anticancer drug.

## 1. Introduction

Human embryonic stem cells (hESCs) provide an inexhaustible source of specialized cells to hold the greatest promise for therapeutic perspectives and basic research of human development [1,2]. hESCs are highly sensitive to DNA-damaging agents, which induce DNA repair mechanisms following genomic insult [3]. In eukaryotic cells, gene activation or repression (and, consequently, the execution of the differentiation program) is highly influenced by the structure of the chromosomal DNA, which is associated with histones and nonhistone HMG proteins, in particular, the HMGB-type proteins [4,5,6]. The majority of HMGB research has focused on the evolutionarily highly conserved HMGB1 and HMGB2 proteins, which are detectable at a high level in all types of embryonic and adult cells [7,8]. *HMGB1* and *HMGB2* are expressed in human inner cell mass (ICM, [9]) and are highly upregulated in human ESCs [8,10]. *HMGB2* expression is selectively downregulated during embryonic development, and the protein is expressed only in certain tissues in adult organisms. *HMGB1* continues to be expressed in all embryonic and adult cells (reviewed in [4,11,12]). The critical roles of HMGB1/2 proteins in cell survival, embryonal neurogenesis, regulation of cell proliferation, differentiation, and telomere biology have been previously demonstrated [4,10,13,14,15,16,17,18].

Telomerase is a reverse transcriptase that counteracts the erosion of chromosome termini in most mammalian cells and plays a pivotal role in regulating the proliferation and senescence of normal somatic cells and cancer cells (reviewed in [19] and refs. therein). The absence of telomerase activity results in telomere shortening, and critically short telomeres trigger the DNA damage response, followed by senescence or cell death [19,20]. Telomerase activity is downregulated in most somatic cell types, but 90% of malignant tumors reactivate telomerase as a prerequisite for their unlimited growth potential. Telomerase activity is widespread in early human development and consistent with this, human embryonic stem cells (hESCs) have robust telomerase activity to maintain telomere length and cellular immortality (reviewed in [21,22]). Telomerase is also upregulated in human cancer cells after exposure to the anticancer drug etoposide [23,24]. It has previously been shown that undifferentiated hESCs are much more sensitive to etoposide-induced apoptosis than their more differentiated progeny, and that p53 is required for etoposide-induced apoptosis of the cells [25]. Etoposide can induce DNA damage-induced apoptosis in hESCs via the activation of both caspase-3 and transcription-dependent functions of p53 [25]. The combination of telomerase inhibition with apoptosis-inducing drug treatments was shown to increase cell death compared to drug treatment alone ([26,27] and refs. therein). Our recent findings on the distinct modulation of cellular activity of telomerase by HMGB1 and HMGB2 [10,17,28] could be a starting point for developing new approaches aiming at the promotion of cell death in drug-treated cancer cells. 

The aim of this study was to study the impact of deregulated expression of *HMGB1* and/or *HMGB2* on stemness, cell cycle distribution, apoptosis, telomere integrity, and telomerase activity in hESCs and their differentiated derivatives, neuroectodermal cells (hNECs), treated with the anticancer drug etoposide.

## 2. Materials and Methods

### 2.1. Cloning of Inducible HMGB-shRNA Constructs

Sequences for *HMGB1* and/or *HMGB2* for knockdown (KD) by vector-based shRNA interference were designed using the Smith‒Waterman algorithm and BLAST sequence alignments (NCBI, Bethesda, MD, USA). The prepared inducible HMGB-shRNA constructs were then tested to determine which had the most efficient *HMGB* silencing. The impact was investigated using at least two different shRNA *HMGB* constructs for a given *HMGB* gene along with “scrambled” shRNA controls previously tested in several human cell lines [10,11,12,13,14,15,16,17,18,19,20,21,22,23,24,25,26,27,28,29]. The sense and antisense strands containing the shRNA-expressing sequence targeting human *HMGB1*, *HMGB2*, or both *HMGB1* and *HMGB2* were annealed and ligated into the BglII/HindIII-linearized pSuperior-puro vector (http://www.oligoengine.com), as detailed in [10].

### 2.2. Generation of Stably Transfected hESCs with Inducible HMGB-shRNA Constructs

The undifferentiated hESCs (line CCTL14) used in this study have previously been thoroughly characterized for the expression of pluripotency transcription factors and their ability to differentiate, as described [30]. Mouse embryonic fibroblasts (MEF)-conditioned media (MEF-CM) were used to culture hESCs under feeder-free conditions on Matrigel^TM^ hESC-qualified matrix (BD Biosciences, San Jose, CA, USA) as described in [10]. The procedures used for preparation of stably transfected and inducible hESCs capable of *HMGB1* and/or *HMGB2* KD are detailed in [10]. Induced expression of the shRNAs targeting *HMGB*s was initiated upon the addition of 0.1 μg/mL of doxycycline (Dox) for 5–10 days. *HMGB1* and/or *HMGB2* KD was verified by Western blotting and/or immunofluorescence microscopy.

### 2.3. Human Neuroectodermal Lineage Commitment from hESCs

The establishment of neuroectodermal cells (hNECs) capable of rosette structure formation was detailed in our previous paper [10]. Briefly, hESCs were seeded at a density of 2‒5 × 10^3^ cells/cm^2^ on culture plates precoated with Matrigel™ in the presence of neural differentiation media (DMEM/F12/Neurobasal 1:1, N2, B27 (Invitrogen, Carlsbad, CA, USA), 2 mmol/L L-glutamine (all media components from Invitrogen), 1× MEM nonessential amino acids, 1% penicillin‒streptomycin and (composition described in [10,17]), and 5% CO_2_ in a humidified atmosphere at 37 °C. After 5 days of culture, a specific neuroectodermal cell morphology emerged, and neuronal rosettes appeared after 8‒10 days. Two distinct scenarios for the differentiation of hESCs into neuroectodermal cells (hNECs) were examined. *HMGB* silencing was performed before (Scenario A) or after (Scenario B) differentiation of hESCs into the hNECs, which was initiated as detailed in [10,17].

### 2.4. Crystal Violet Staining

Growth curves of hESCs or hNECs in the presence or absence of etoposide were determined using crystal violet staining, as detailed in [10,17].

### 2.5. Western Blotting

Cells were resuspended in lysis buffer (50 mM Tris-HCl (pH 6.8) containing 10% glycerol and 1% sodium dodecyl sulfate (SDS)), and protein concentrations were determined using the DC Protein Assay Kit (Bio-Rad, Hercules, CA, USA). Equal amounts of proteins from cellular lysates were loaded onto 10‒12.5% SDS-PAGE (sodium dodecyl sulphate-polyacrylamide gel electrophoresis) gels and electrophoresis was carried out as detailed in [10,17]. Resolved proteins were then transferred onto Immun-Blot^®^ PVDF Membranes (Bio-Rad: 1620177) using a wet-transfer system operated at 100 V for 2 h. The membranes were washed with Tris-buffered saline containing Tween-20 (TBST) and incubated with primary and HRP-conjugated secondary antibodies, as detailed in [10,17]. Detection of proteins on the membranes was performed using SuperSignal™ West Dura Extended Duration Substrate reagent (Thermo Fisher Scientific, Waltham, MA, USA), followed by visualization using the FujiFILM Luminescence image analyzer LAS-3000 instrument (LifeSciences, Irvine, CA, USA).

### 2.6. Cell Cycle Assay by Flow Cytometry

Cultured hESCs and hNECs were dissociated using TrypLE Select (Thermo Fisher Scientific, USA) and Accutase (Sigma-Aldrich, St. Louis, MO, USA), respectively; the pellets were resuspended in cold 1× PBS and counted with a hemocytometer. After fixation in ice-cold 70% ethanol (*v*/*v*) for 1 h, cells were washed in ice-cold PBS and incubated with RNaseA (final concentration 0.02 mg/mL; Boehringer Ingelheim, Germany) at 37 °C for 30 min. Propidium iodide was then added (40 μg/mL in PBS; Sigma-Aldrich, USA) for 10 min. Finally, the data were acquired using a FACS Canto II (BD Biosciences, San Jose, CA, USA). The distribution of cells in different cell cycle phases was measured using FlowJo^TM^ software (http://www.flowjo.com).

### 2.7. Immunocytochemistry

Cells were washed with PBS, fixed with 4% paraformaldehyde in PBS pH 7.4 for 20 min at RT, permeabilized with PBS containing 0.1% Triton X-100 for 20 min, and blocked with 1% bovine serum albumin in PBS for 1 h. The cells were then washed three times with PBS and incubated with diluted primary antibodies at 4 °C overnight. The washed cells were incubated with the appropriate AlexaFluor-conjugated secondary antibodies (as detailed in [10,17]) for 2 h at RT. Cell nuclei were counterstained with 4,6-diamidino-2-phenylindole (DAPI) (Sigma-Aldrich).

### 2.8. Confocal Microscopy

For fluorescence staining, control hESCs and cells with downregulated *HMGB1* and/or *HMGB2* expression were cultured on Matrigel-coated sterilized coverslips placed into wells of a 24-well tissue culture plate. Cells were maintained under feeder-free conditions using either CM or hNEC-induced neural media in the presence of Dox for five or 12 days [10,17]. After washing the coverslips with cultured cells with PBS (1×), the cells were fixed with cold 100% methanol for 10 min at ‒20 °C, and permeabilized with 0.2% Triton X-100 in PBS (Sigma Aldrich) for 30 min. After washing the cells twice with PBS (1×), the cells were blocked for 30 min in a blocking solution composed of PBS supplemented with 0.1% Tween-20 (PBST) and 1% BSA. Then, cells were incubated with primary antibodies (diluted 1:50‒300 in PBST) for overnight at 4 °C, followed by incubation with secondary antibodies (diluted 1:500 in PBST) conjugated to Alexa Fluor 488 and Alexa Fluor 568 (Thermo Fisher) at room temperature for 1 to 2 h. Finally, cells were labeled with DAPI for 3‒5 min, and coverslips were mounted on slides using Mowiol (Calbiochem, San Diego, CA, USA), allowed to dry, and fluorescence images of the cells were taken using an Olympus Fluoview FV10i confocal laser scanning microscope (Olympus Corporation, Tokyo, Japan). CellProfiler, free open-source software (McQuin, 2018 #4686}), was applied to measure and analyze all of the resulting images from the confocal microscope [31].

### 2.9. Annexin V-FITC/Propidium Iodide Double-Staining Assay

While Annexin V is used to determine whether cells have undergone apoptosis, propidium iodide (PI) is used to detect necrotic or late apoptotic cells. An Annexin V/PI double staining kit (ApoFlowExFITC Kit, cat. No: ED7044, EXBIO) was used in flow cytofluorimetric analyses according to the manufacturer’s instructions. Briefly, cells (floating and attached, untreated or treated with etoposide) were collected and washed twice with ice-cold PBS; then, the cell pellet was resuspended by flicking in 100 µL 1× binding buffer and the cell density adjusted to 2‒5 ×10^5^ cells/mL. Subsequently, 5 µL Annexin V-FITC and 5 µL propidium iodide were added and incubated in the dark at RT for 15 min. The prepared cells (viable, apoptotic, and necrotic) were immediately quantified by flow cytometry (FACS Canto II; BD Biosciences) with at least 10,000 events for each sample and analyzed using FlowJo software. The data generated by flow cytometry were plotted in two-dimensional dot plots in which they could be divided in four categories corresponding to Q1 (Annexin V−/PI+), dead cells; Q2 (Annexin V+/PI+), late apoptotic and necrotic cells; Q3 (Annexin V−/PI-), viable cells; Q4 (Annexin V+/PI-), early apoptotic cells.

### 2.10. Cell Cycle and Apoptosis of Etoposide-Treated Cells

hESCs or hNECs (control cells or cells after *HMGB* silencing) were incubated with 3.4 μM etoposide for 3, 12, or 24 h at 37 °C. After treatment, adherent and floating cells were harvested by enzymatic dissociation, washed once with PBS, and pelleted by centrifugation, as detailed for untreated cells in [10]. Prior to analysis by FACS Canto II, the cells were labeled using either propidium iodide for cell cycle distribution or an Annexin V/PI Apoptosis Detection kit (ApoFlowExFITC Kit, EXBIO) according to the manufacturer’s instructions. Subsequently, the data about the distribution of cell cycle phases and apoptosis induction were obtained using FlowJo software. 

### 2.11. Image Acquisition and Analysis

Microscopy images of cells grown in 24-well plates were recorded with an automated ImageXpress XL (Molecular Devices, Sunnyvale, CA, USA). To cover a maximal surface area of the wells, 49 images were taken for each well in a DAPI channel. To quantify the neural rosette structures using CellProfiler software, the images were first smoothed using a Gaussian filter, and neural rosettes were then detected using an Otsu thresholding method, as indicated in [10,17]. The percentage of the image area covered by the neural rosettes was subsequently determined.

### 2.12. Determination of Telomerase Activity by TRAP Assay

Cellular lysates for the estimation of telomerase activity were prepared from cells that had been lysed in CHAPS detergent (200 μL per 10^6^ cells) on ice for 30 min, followed by centrifugation (1200× *g*) for 20 min. Telomerase activity was determined in cellular lysates (24 ng of total proteins) by the qPCR TRAP (telomeric repeat amplification protocol) assay using primers as detailed in [17]. Briefly, samples were incubated at 37 °C for 30 min, followed by initial denaturation at 94 °C for 3 min and three subsequent cycles (95 °C/30 s, 55 °C/60 s, and 72 °C/30 s), and a final 40 cycles (94 °C/30 s, 55 °C/30 s and 72 °C/30 s). The fluorescence signal was acquired at 55 °C during the course of the final 40 PCR cycles. TRAP analyses were performed in quadruplicate and independently verified in at least three other experiments. All qPCR analyses were performed using Rotorgene 6000 (Rotor-Gene 6000 Series Software 1.7).

### 2.13. Statistical Analysis

All quantitative data are presented as the mean with standard deviation (SD). Statistical analysis among groups was carried out by means of a one-way ANOVA test, followed by Tukey’s multiple comparison test post hoc analysis or by Bonferroni post hoc test. In some cases, data were analyzed using a *t*-test. For all statistical analyses, *p* > 0.05 was considered not significant (ns); * *p* < 0.05, ** *p* < 0.01, *** *p* < 0.001, and **** *p* < 0.0001. All data were analyzed using GraphPad Prism version 5.0 for Windows (GraphPad Software, La Jolla, CA, USA). All experiments were carried out in triplicate.

## 3. Results

### 3.1. Pluripotent hESCs with Downregulated HMGB1/2 Expression Retain Stemness and Pluripotent State

Although our knowledge regarding HMGB1 and HMGB2 as nuclear transcription regulators has steadily advanced over the last decade, their functional roles and regulatory activities in hESCs and during the early stages of development remain unclear. In this paper, we have investigated the impact of *HMGB1* and/or *HMGB2* knockdown (KD) on the stemness, cell cycle distribution, apoptosis, telomere integrity, and telomerase activity in hESCs and their differentiated derivatives, neuroectodermal cells (hNECs), in the presence or absence of the anticancer drug etoposide.

**Figure 1:**
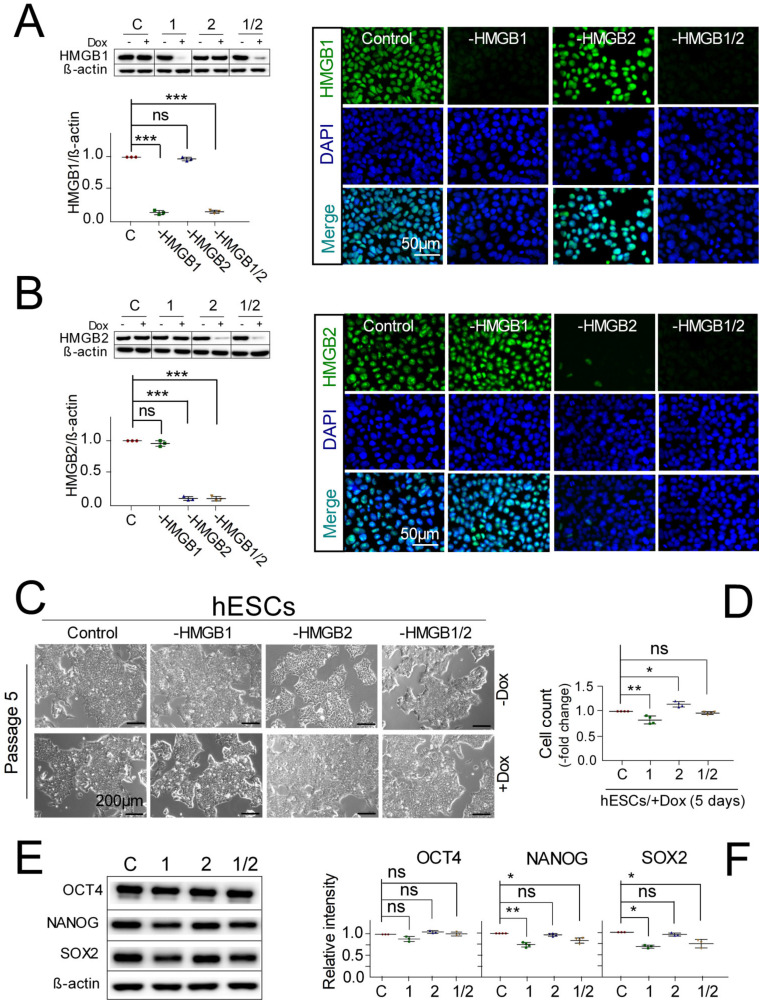
The morphology of human embryonic stem cells (hESCs) remains unchanged after knockdown of *HMGB1* and/or *HMGB2* while maintaining self-renewal and pluripotency in culture. (**A** and **B**) Left panels: The level of Dox-inducible *HMGB1* and *HMGB2* knockdown in hESCs as determined by Western blotting. Bar graphs represent the quantification of Western blots. All data on *HMGB* expression were normalized to β-actin. Knockdown of *HMGB1* or *HMGB2* in control hESCs (empty vector transfected) was set as 1. Right panels: Immunostaining of HMGB1 and/or HMGB2 in stably transfected hESC clones with inducible *HMGB* knockdown. Anti-HMGB1 or anti-HMGB2 (green) antibodies were used. DNA was stained with 4,6-diamidino-2-phenylindole (DAPI, blue). (**C**) Normal morphology of hESCs in the absence (−) or presence (+) of doxycycline (Dox) under standard culture media for 5 passages. (**D**) Relative number of hESCs upon *HMGB1* and/or *HMGB2* knockdown (results are normalized to empty vector-transfected cells). Levels of the pluripotency markers OCT4, NANOG, and SOX2 upon *HMGB1* and/or *HMGB2* knockdown in hESCs, as revealed by Western blotting (**E**) or densitometric quantification (**F**) of the Western blots. Description: “C”, cells transfected with empty vector; “−1”, cells with *HMGB1* knockdown; “−2”, cells with *HMGB2* knockdown; “−1/2”, cells with *HMGB1* and *HMGB2* knockdown. All calculations were normalized against β-actin. The expression level of pluripotency markers in the control (empty vector transfected) cells was set as 1. Data represent the mean and standard deviation (SD) of three independent experiments. Data were analyzed using Tukey’s multiple comparison test (*p* > 0.05 = not significant, ns; * *p* < 0.05; ** *p* < 0.01; *** *p* < 0.0001). Scale bars = 50, 200 μm.

hESCs capable of Dox-inducible *HMGB1* and/or *HMGB2* KD were generated using previously described methods [10,17]. The addition of Dox to the culture media of pluripotent human embryonic stem cells (hESCs) confirmed that *HMGB1* or *HMGB2* expression was reduced by ~90‒95% in KD cells, on average, as determined by Western blotting (Figure 1A,B, left panels). As Dox can affect cell survival/self-renewal and other cellular properties, we have used the lowest possible concentration of Dox (0.1 μg/mL) in our experiments to reduce its impact on hESCs while still achieving the highest *HMGB* knockdown. Knockdown of *HMGB1* and/or *HMGB2* in hESCs was also verified by immunostaining of the cells (Figure 1A,B, right panels). Importantly, *HMGB1* and/or *HMGB2* KD did not alter the morphology of undifferentiated hESCs during 4 weeks of culture (passage 5 in Figure 1C). Compared to somatic cells, pluripotent hESCs proliferate much faster due to a very short overall cell cycle [32]. Therefore, we were also interested to see whether *HMGB* silencing could change the proliferation rate of hESCs. As shown in Figure 1D, cultivation of hESCs possessing a *HMGB1* shRNA construct with 0.1 μg/mL Dox for 5 passages displayed a decreased number of cells relative to control cells. Conversely, cells with *HMGB2* KD increased in proliferation, whereas double *HMGB1/2* KD exhibited a similar proliferation rate to the control cells (Figure 1D).

To further examine the impact of *HMGB1* and/or *HMGB2* KD on the pluripotency of human embryonic stem cells, we performed Western blotting of the pluripotent stem cell markers OCT4, NANOG, and SOX2. hESC lines with inhibited *HMGB*s continued to self-renew and expressed common pluripotency markers OCT4, NANOG, and SOX2 (Figure 1E). Densitometric quantification of Western blots from Figure 1E revealed that *HMGB2* KD had no impact on the levels of pluripotency proteins, while *HMGB1* and *HMGB1/2* KD downregulated the levels of NANOG and SOX2, but not OCT4 (Figure 1F). However, the decreased levels of NANOG and SOX2 observed upon *HMGB1* or *HMGB1/2* double KD (Figure 1E,F) had no impact on the pluripotency of hESCs, as revealed using a teratoma formation assay, in which no tangible deviation from the normally differentiated teratoma morphology was demonstrated [10]. Thus, both control hESCs and the cells upon *HMGB1* and/or *HMGB2* KD displayed a similar potential to differentiate toward all three germ layers in vivo, revealing no impact of the knockdown of *HMGB*s on hESC stemness and pluripotent state [10].

### 3.2. Distinct Impact of HMGB1/2 KD on Survival and Neuroectodermal Lineage Commitment

Recent findings suggest that HMGB proteins could differentially affect neurogenesis through the regulation of neural stem cell (NSC) proliferation and survival [13,33,34]. Previous studies demonstrated the involvement of SOX2 and PAX6 (markers of stem/progenitor cell proliferation and neurogenesis) in the developing human nervous system [35] and the derivation of human neural stem cells (hNSCs) from hESCs [36]. The radially organized columnar epithelial cells termed “neural rosettes”, derived from hESCs, display a novel stem cell state in the progression of hESCs toward differentiated neural commitments.

**Figure 2:**
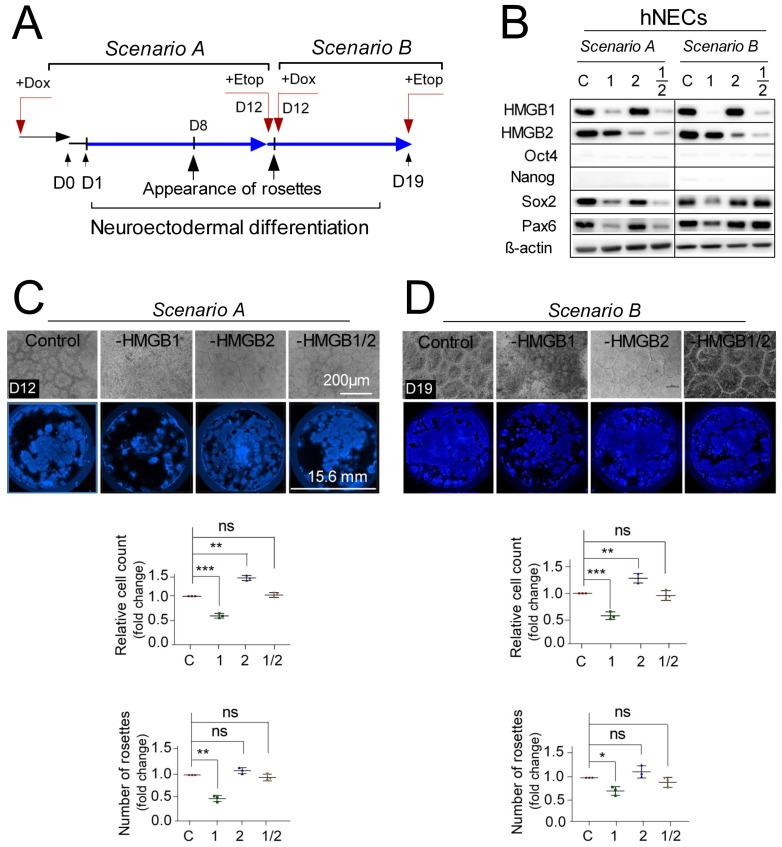
The distinct impact of *HMGB1*/*2* silencing on the cell survival and differentiation efficiency of neuroectodermal cells. (**A**) Two scenarios for the differentiation of hESCs into the neuroectodermal lineage. Scenario A: Undifferentiated hESCs were treated with doxycycline (Dox) for at least 3 days and subsequently induced to differentiate (D1) into neuroectodermal cells for 12 days in the continuous presence of Dox in differentiation media. On day 12 (D12), the cells were harvested for analysis. Scenario B: Undifferentiated hESCs were induced to differentiate to human neuroectodermal cells (hNECs) for 12 days. At that point (D12), Dox was added to the differentiation media, and the hNECs were allowed to grow for another 7 days in the presence of Dox. On day 19 (D19), the cells were harvested for analysis. (**B**) Expression of the pluripotency markers OCT4 and NANOG and the differentiation-associated proteins SOX2 and PAX6 as determined by Western blotting of cellular lysates from hNECs prepared either by Scenario A or Scenario B. Equal loading of samples was verified using the anti-β-actin antibody. Description: “C”, cells transfected with empty vector; “1”, cells upon *HMGB1* knockdown; “2”, cells upon HMGB2 knockdown; “1/2”, cells upon *HMGB1* and *HMGB2* knockdown. (**C**,**D**) Morphology of neural rosettes as visualized by phase contrast microscopy using an Image Xpress XL automated microscope (Molecular Devices, San Jose, CA, USA; top panels). Bar graphs indicate the relative number of hNECs and quantification of rosette structure formation upon *HMGB1* and/or *HMGB2* knockdown (lower panels; results are normalized to empty vector-transfected cells). Data were analyzed using Tukey’s multiple comparison test (*p* > 0.05 = not significant, ns; * *p* < 0.05; ** *p* < 0.01; *** *p* < 0.0001) and represent the mean and SD of three independent experiments. Scale bars = 50, 200 μm.

In order to investigate whether *HMGB1* and/or *HMGB2* KD are associated with the regulation of neuroectodermal lineage commitment and how the loss of the *HMGB1* and/or *HMGB2* could affect the cell cycle/apoptosis/telomerase activity of hESCs and their derivatives (neuroectodermal cells) treated with the anticancer drug etoposide, we used two distinct protocols to differentiate human ESCs into neuroectodermal cells (referred to as hNECs). Scenario A was used to assess the ability of hESCs to differentiate in the absence of HMGB proteins. As shown in Figure 2A (Scenario A), *HMGB1* and/or *HMGB2* expression was continuously inhibited by the addition of Dox from at least one week before the initiation of differentiation (D1, the time at which the neurodifferentiation media were added) until day 12 of differentiation. Scenario B was used to assess the impact of deregulated *HMGB* expression on already differentiated hNECs. In Scenario B, differentiation was initiated under normal conditions, and *HMGB* silencing was initiated upon addition of Dox for only 7 days, beginning on day 12 of differentiation (Figure 2A, Scenario B). Western blotting confirmed that the desired downregulation of *HMGB1* and/or *HMGB2* expression was achieved in both differentiation scenarios (Figure 2B). The pluripotency markers OCT4 and NANOG became undetectable upon differentiation, regardless of the HMGB status and the differentiation scenario. While PAX6 and SOX2 were detectable in hNECs regardless of the *HMGB* KD, these markers were significantly decreased upon *HMGB1* KD using both differentiation scenarios (Figure 2B). *HMGB1* KD in hNECs resulted in a two-fold decrease in the number of the differentiated cells, regardless of the differentiation scenario (Figure 2, panels C/D). Correspondingly, the formation of rosettes in hNESCs upon *HMGB1* KD was also significantly decreased by ~65% and ~30% in Scenarios A and B, respectively. No impact of *HMGB2* KD on the formation of rosettes in hNESCs was observed in our experiments (Figure 2C,D).

### 3.3. Pluripotent hESCs Exposed to Etoposide are more Susceptible to Cell Death than the hESC-Derived Progenitor Cells

It was previously revealed that undifferentiated human embryonic stem cells were much more sensitive to etoposide-induced apoptosis than their more differentiated progeny, and that p53 was required for etoposide-induced apoptosis of the cells [25]. The aim of our subsequent experiments was to determine the impact of deregulated expression of *HMGB1* and/or *HMGB2* on the cell survival and cell cycle of hESCs and progenitor cells (hESC-derived neuroectodermal cells, hNECs).

**Figure 3:**
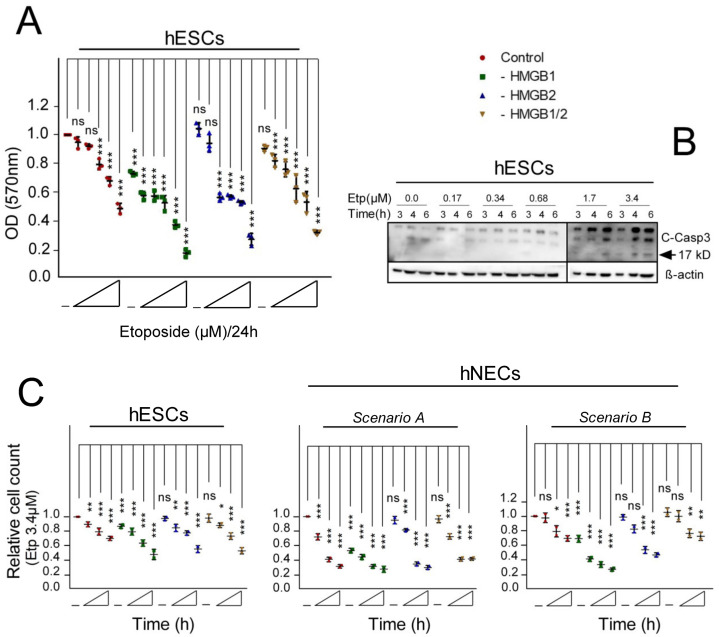
*HMGB1/2* knockdown (KD) can differentially modulate the cell viability of hESCs and hNECs treated with the anticancer drug etoposide. (**A**) hESCs were treated with increasing concentrations of etoposide (0.17, 0.68, 1.7, 3.4, and 17 μM) for 24 h, and the growth curves were recorded by measuring the absorbance of crystal violet at 570 nm (results were normalized against untreated control cells, which were set as 1). (**B**) Cleavage of caspase-3 in etoposide-treated hESCs as revealed by Western blotting. Cellular lysates from control (empty vector-transfected) hESCs were treated with increasing concentrations of etoposide (0.17‒3.4 μM) for 3, 4, or 6 h. Equal loading of samples was verified using anti-β-actin antibody. (**C**) Relative cell count of 3.4 μM etoposide-treated hESCs and hNECs (prepared by Scenarios A or B) was measured using the Cedex automated cell counting system (Roche Diagnostics, Mannheim, Germany) at 0, 6, 12, and 24 h (left to right). Data were analyzed using Bonferroni post hoc test and represent the mean and SD of three independent experiments (* *p* < 0.05; ** *p* < 0.001; *** *p* < 0.001).

To determine the optimal concentrations of etoposide for subsequent experiments, the induction of apoptosis was monitored by assessing the activity of caspase-3, one of the central executioners of apoptosis, in hESC cells (Figure 3B). While there were no morphological signs of damaged apoptotic or necrotic cells (data not shown), cleavage of caspase-3 was the most prominent at 3.4 μM etoposide in the course of treatment (Figure 3B); consequently, we decided to use this etoposide concentration for most of the subsequent experiments. Etoposide at 3.4 μM could decrease cell growth of both undifferentiated hESCs and differentiated hNECs by ~2-fold in a time-dependent manner (Figure 3C). Loss of *HMGB1* and, to a lesser extent, *HMGB2*, further promoted the cell growth inhibition in the etoposide-treated hESCs (Figure 3C). We have observed that the neuroectodermal cells (hNECs) prepared according to Scenario A were more sensitive to 3.4 μM etoposide than the cells prepared according to Scenario B (Figure 3C). The sensitivity of hNECs, irrespective of the differentiation protocols, was further increased in cells with deregulated expression of *HMGB1* or *HMGB2*.

### 3.4. Cell Cycle Progression in HMGB KD hESCs and hNECs Treated with Etoposide

The distribution of cell cycle phases appears to be distinct in human embryonic stem cells compared to differentiated cells. Pluripotent hESCs have a quick cell cycle with an abbreviated G1 phase [32]. Further studies revealed that hESCs could initiate endoderm/mesoderm gene expression and neuroectoderm markers only during the early and late G1 phase, respectively [37], whereas hESCs responded poorly to differentiation signals in the G2/S/M phases of the cell cycle [38]. Activation of cell death by etoposide is cell cycle dependent due to the suppression of topoisomerase II activity, which is essential for the progression through S phase [39]. As the effect of some anticancer drugs is associated with different cell cycle phases [40], we have determined the impact of etoposide on cell cycle progression and sub-G1 arrest in control and *HMGB1* and/or *HMGB2* KD cells.

Time-course monitoring of the cell cycle distribution in etoposide-treated hESCs (Figure S1) revealed that 3.4 µM of etoposide caused significant accumulation of the cells in G0/G1 phase and sub-G1 peaks (an indicative of apoptosis), with a concomitant decrease of the cells in G1 phase after 3‒24 h. A significant increase in the S phase population was observed in hESCs or hNECs prepared by Scenario A (but not by Scenario B) soon after etoposide treatment (Figure S1: compare panels B and H,I). The increase in the S phase population was abolished at the onset of apoptotic cell death upon prolonged etoposide treatment for 12‒24 h. The populations of cells in the sub-G1 phase were similar in control (empty vector-transfected) cells and *HMGB1/2* KD hNECs (Figure S1, panels E,H) for 0‒24 h.

In summary, our results indicate that etoposide treatment of the *HMGB1* KD hESCs or hNECs resulted in an increased percentage of sub-G1 cells (Figure S1B). While a higher percentage of sub-G1 cells was also observed in etoposide-treated *HMGB1* KD hNECs (irrespective of the differentiation protocol; Figure S1, panels E,H), an increase in the percentage of sub-G1 cells was observed upon 3 h etoposide treatment of the *HMGB2* KD hNECs prepared according to Scenario B, but not in the *HMGB2* KD hNECs prepared according to Scenario A (Figure S1, panels E,H) or in the *HMGB2* KD hESCs (Figure S1B). Thus, etoposide can suppress the growth of hESCs and hNECs by hindering cell cycle progression and induction of sub-G1 arrest in a time- and HMGB-dependent manner.

### 3.5. Apoptosis of Etoposide-Treated hESCs is Enhanced upon HMGB1 KD

Apoptosis (programmed cell death) is essential for proper homeostatic maintenance and survival in multicellular organisms. Previous reports have illustrated that hESCs are more vulnerable to rapid apoptosis upon genotoxic stress compared to ordinary somatic cells [3,25,41]. *HMGB1* KD was reported to increase drug sensitivity in cancer cells, and it was assumed that HMGB1 plays transcription-dependent (such as regulation of Bcl-2 family protein expression) and transcription-independent roles (such as regulation of autophagy and p53 location) in the regulation of apoptosis (reviewed in [42]). However, the contribution of HMGB1/2 proteins to the apoptosis of hESCs and progenitor cells mediated by treatment of the cells with anticancer drugs is unclear.

**Figure 4:**
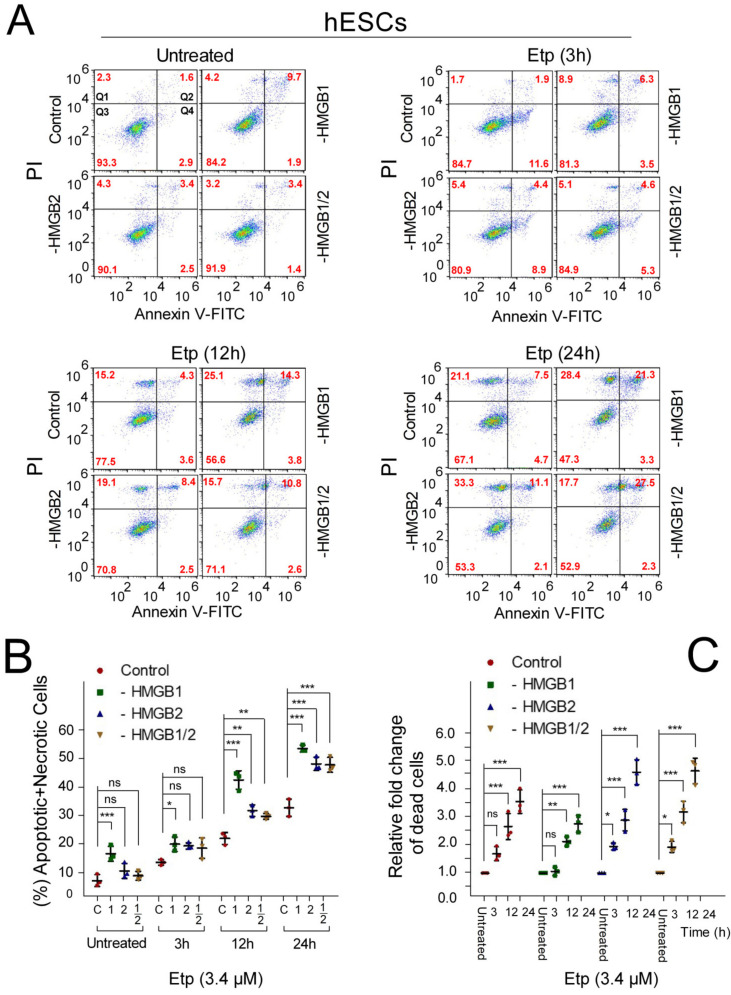
Distinct impact of *HMGB* KD on apoptosis of hESCs treated with etoposide. (**A**) Representative dot plots showing Annexin V/propidium iodide (PI) binding assays in etoposide-treated hESCs upon *HMGB1* and/or *HMGB2* KD, as analyzed by flow cytometry. Q1 (Annexin V−/PI+), dead cells; Q2 (Annexin V+/PI+), late apoptotic and necrotic cells; Q3 (Annexin V−/PI−), viable cells; Q4 (Annexin V+/PI−), early apoptotic cells. Values in red represent percentages of cells in Q1–Q4. (**B**) Percentage of apoptotic + necrotic cells in hESCs from (**A**). (**C**) Relative fold change of total cell death among populations of control or *HMGB* KD hESCs compared to untreated cells. Error bars represent the mean and SD of three independent experiments. Data were analyzed using either Bonferroni post hoc test for the (percentage of apoptotic + necrotic cells) or Tukey’s multiple comparison test for analysis of the relative fold change of dead cells (*p* > 0.05 = not significant, ns; * *p* < 0.05; ** *p* < 0.01; *** *p* < 0.001). “C”, cells transfected with empty vector; “HMGB1”, cells upon *HMGB1* knockdown; “HMGB2”, cells upon *HMGB2* knockdown; “HMGB1/2”, cells upon *HMGB1* and *HMGB2* knockdown. “1”, cells upon *HMGB1* knockdown; “2”, cells upon *HMGB2* knockdown; “1/2”, cells upon *HMGB1* and *HMGB2* knockdown.

Here, we have used flow cytometry and analyzed the impact of *HMGB* KD on apoptosis in Annexin V/PI-labeled hESCs (Figure 4) upon incubation of the cells with 3.4 μM etoposide for different periods of time (0‒24 h). *HMGB1* KD resulted and a ~2-fold increase in the percentage of apoptotic/necrotic cells in etoposide-untreated hESCs (Figure 4B), in agreement with previous reports published on other cell lines [43]. A high increase in the percentage of apoptotic/necrotic cells was observed in the etoposide-treated cells after 12 h, although a small increase in apoptotic/necrotic cells was detectable for all etoposide incubation times (Figure 4B). While *HMGB2* or *HMGB1/2* KD did not affect the percentage of apoptotic/necrotic cells in etoposide-untreated hESCs, treatment of the *HMGB2* KD hESCs with etoposide for 12‒24 h resulted in a concentration-dependent increase in apoptotic (Annexin V-positive cells) and necrotic (PI-positive cells) cell death (Figure 4B). Our findings revealed an important role of both HMGB1 and HMGB2 in sensitizing hESCs for the anticancer drug etoposide.

### 3.6. The Impact of HMGB1/2 Knockdown on Apoptosis of Etoposide-Treated Progenitor Cells Depends on the Differentiation Protocol of hESCs

**Figure 5:**
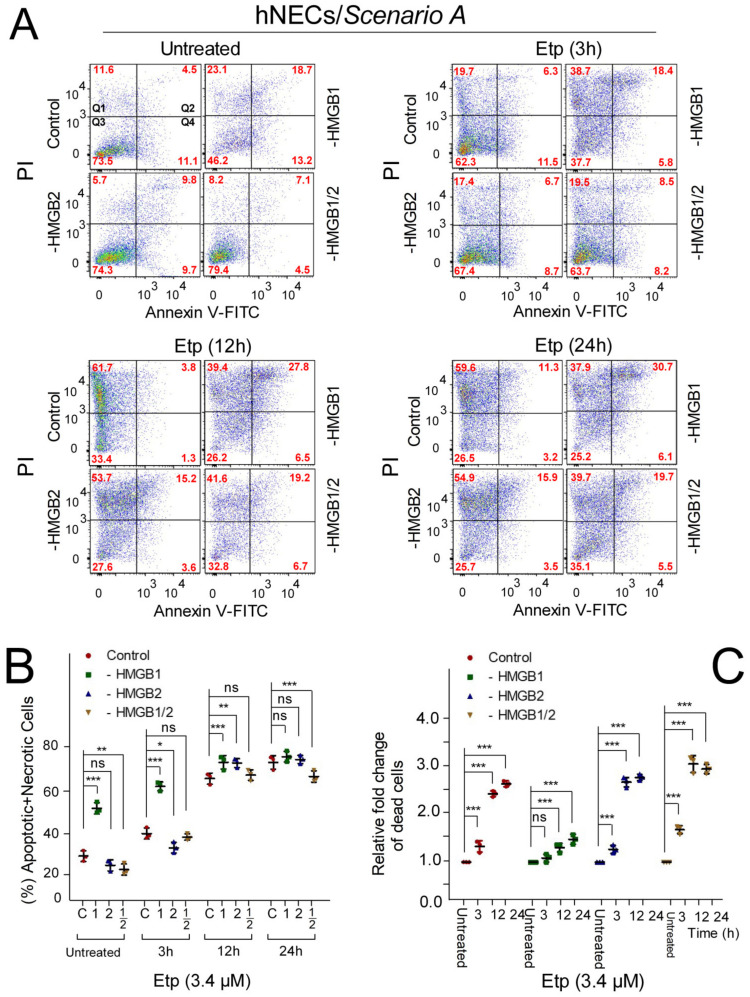
The impact of *HMGB1*/*2* KD on the apoptosis of etoposide-treated hNECs prepared according to Scenario A. (**A**) Representative dot plots showing Annexin V/propidium iodide (PI) binding assays in hNECs upon *HMGB1* and/or *HMGB2* KD, followed by etoposide treatment for 0‒24 h, as analyzed by flow cytometry. (**B**) Percentage of apoptotic/necrotic cells in hESCs from (**A**). (**C**) Relative fold change of total cell death among populations of control or *HMGB* KD hNECs (control cells from each of the cell types were set as 1). Error bars represent the mean and SD of three independent experiments. Data were analyzed using either a Bonferroni post hoc test for the (percentage of apoptotic + necrotic cells) or Tukey’s multiple comparison test for analysis of the relative fold change of dead cells (*p* > 0.05 = not significant, ns; * *p* < 0.05; ** *p* < 0.01; *** *p* < 0.001). “C”, cells transfected with empty vector; “HMGB1”, cells upon *HMGB1* knockdown; “HMGB2”, cells upon *HMGB2* knockdown; “HMGB1/2”, cells upon *HMGB1* and *HMGB2* knockdown. “1”, cells upon *HMGB1* knockdown; “2”, cells upon *HMGB2* knockdown; “1/2”, cells upon *HMGB1* and *HMGB2* knockdown.

**Figure 6:**
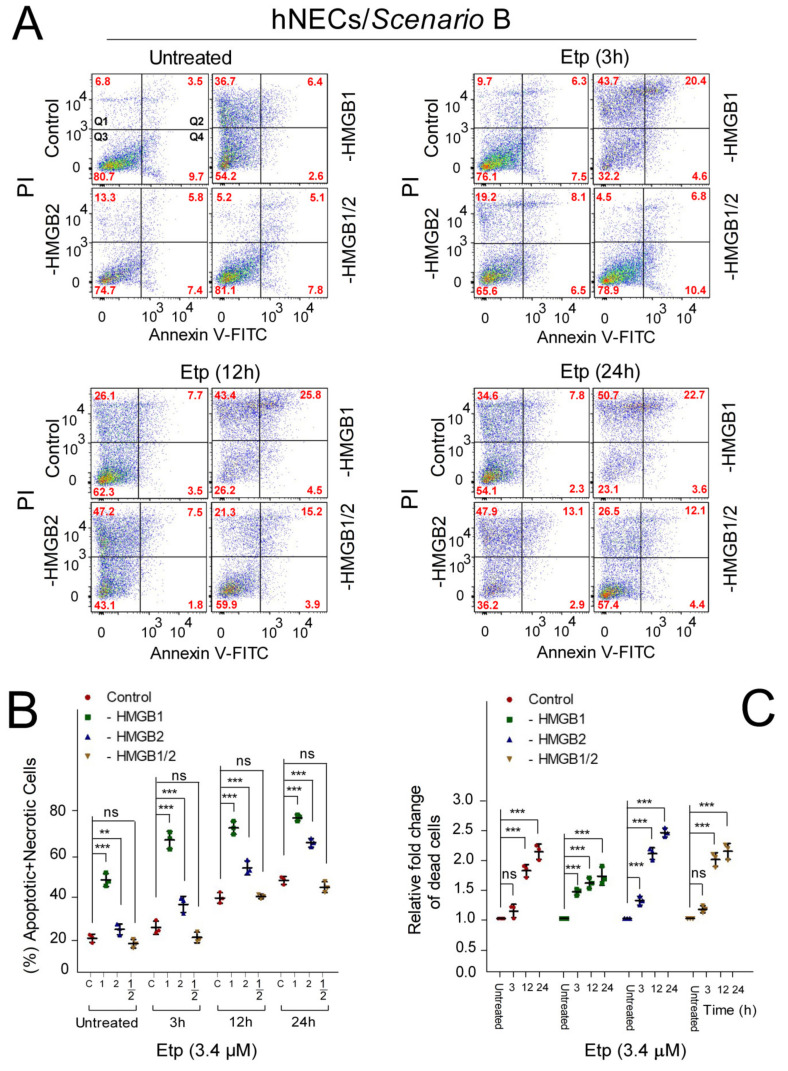
The impact of *HMGB1/2* KD on apoptosis of etoposide-treated hNECs prepared according to Scenario B. (**A**) Representative dot plots showing Annexin V/propidium iodide (PI) binding assays in hNECs upon *HMGB1* and/or *HMGB2* KD, followed by etoposide treatment for 0‒24 h, as analyzed by flow cytometry. (**B**) Percentage of apoptotic + necrotic cells in hESCs from (**A**). (**C**) Relative fold change of total cell death among populations of control or *HMGB* KD hNECs compared to untreated cells. Error bars represent the mean and SD of three independent experiments. Data were analyzed using either Bonferroni post hoc test for the (percentage of apoptotic + necrotic cells) or Tukey’s multiple comparison test for analysis of the relative fold change of dead cells (*p* > 0.05 = not significant, ns; * *p* < 0.05; ** *p* < 0.01; *** *p* < 0.001). “C”, cells transfected with empty vector; “HMGB1”, cells upon *HMGB1* knockdown; “HMGB2”, cells upon *HMGB2* knockdown; “HMGB1/2”, cells upon *HMGB1* and *HMGB2* knockdown. “1”, cells upon *HMGB1* knockdown; “2”, cells upon *HMGB2* knockdown; “1/2”, cells upon *HMGB1* and *HMGB2* knockdown.

We used flow cytometry to analyze the impact of *HMGB* KD on apoptosis in Annexin V/PI-labeled neuroectodermal cells (hNECs) treated with 3.4 μM etoposide for different times (Figure 5). Similarly to the etoposide-untreated *HMGB1* KD hESCs (Figure 4), *HMGB1* KD in etoposide-untreated neuroectodermal cells resulted in a ~2-fold increase in the percentage of apoptotic (Annexin V-positive cells)/necrotic (PI-positive cells) cells, irrespective of the differentiation protocol (Figure 5 and Figure 6). A significant increase in the percentage of apoptotic/necrotic cells was also detected in *HMGB1* KD hNECs treated with etoposide for 3 h (Figure 5 and Figure 6) regardless of the differentiation protocol used (Figure 5 and Figure 6). However, clear differences in the response of *HMGB* KD hNECs to apoptosis were observed between cells differentiated according to Scenarios A and B when treated with etoposide for longer times, i.e., 12 or 24 h (Figure 5 and Figure 6B). While there were no apparent differences in the percentage of apoptotic/necrotic cells in control and *HMGB1* or *HMGB2* KD hNECs differentiated according to Scenario A (Figure 5B), the percentage of apoptotic/necrotic cells in *HMGB1* or *HMGB2* KD hNECs differentiated according to Scenario B was significantly higher than in the control cells (Figure 6B). Interestingly, simultaneous silencing of both *HMGB1* and *HMGB2* resulted in hNECs with a similar percentage of apoptotic/necrotic cells to the control etoposide-treated cells (Figure 6B)

### 3.7. HMGB1 KD Promotes Cell Death Induction in Etoposide-Treated hESCs and hNECs via Activation of Caspase-3 Pathway

One of the most common signaling cascades involved in apoptosis is the activation of enzymes caspases. PARP-1 (a nuclear protein performing many roles in the cell, including central roles in the repair of damaged DNA [44]) is one of several known cellular substrates of caspases. Cleavage of PARP-1 by caspases is considered a hallmark of apoptosis [45]. Activation of caspase-3 is primarily associated with etoposide-induced cell death of hESCs [25].

**Figure 7:**
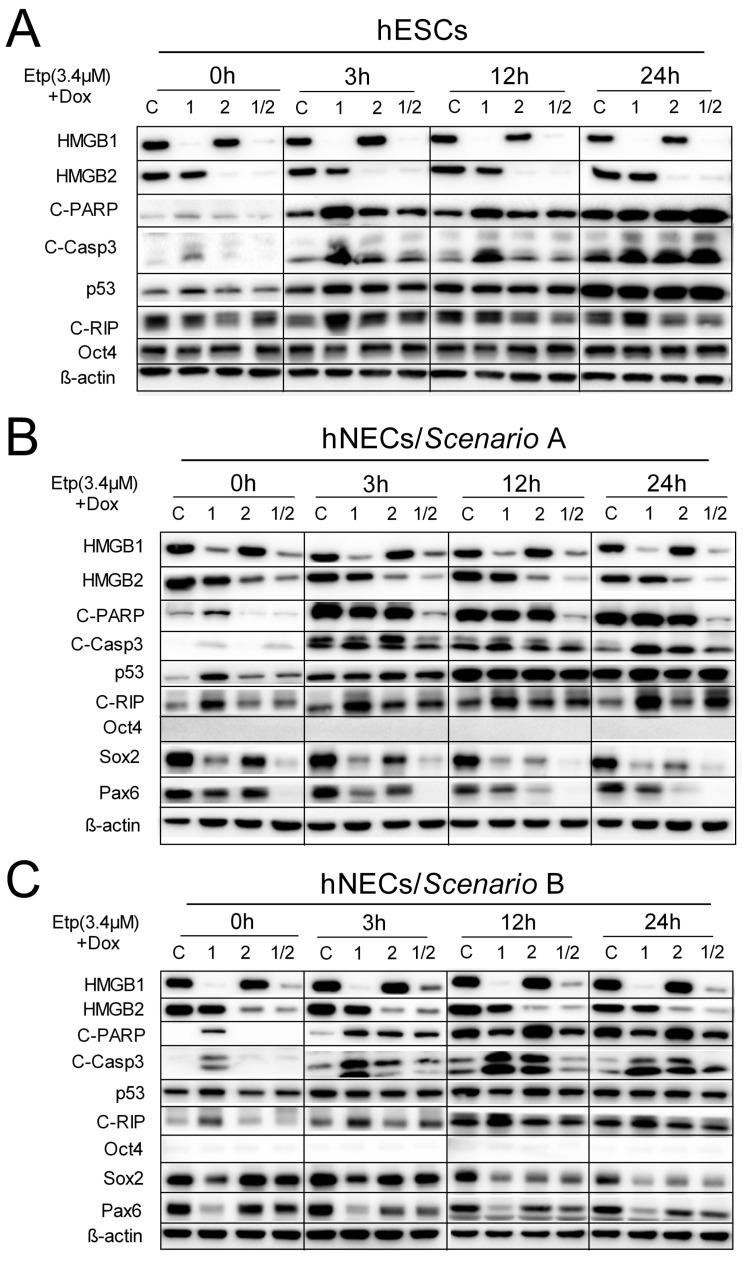
HMGB1 and HMGB2 differentially affect Casp3/PARP cleavage in etoposide-treated hESCs or hNECs. (**A‒C**) Western blotting of total cellular lysates from hESCs and hNECs prepared by Scenario A or B. Equal loading of samples was verified using anti-β-actin antibody. “C”, cells transfected with empty vector; “1”, cells upon *HMGB1* knockdown; “2”, cells upon *HMGB2* knockdown; “1/2”, cells upon *HMGB1* and *HMGB2* knockdown. C-Casp3, cleaved caspase-3; C-PARP, cleaved PARP (poly (ADP-ribose) polymerase); C-RIP, cleaved RIP (receptor-interacting protein kinase).

Here, Western blotting and immunodetection were used to study the impact of *HMGB1* and/or *HMGB2* KD on activation of caspase-3 and PARP cleavage, expression of RIP kinase (a specific marker for necrosis), and transcription factors Oct4, Sox2, and Pax6 in etoposide-treated cells as well as the induction of the tumor suppressor protein p53, which can elicit a response to various DNA-damaging agents (including etoposide) [46]. Caspase-3 and PARP cleavage was already observed in etoposide-untreated *HMGB1* KD hESCs or hNECs (Figure 7) and was further enhanced upon treatment of the cells with etoposide. In agreement with the observed increased percentage of apoptotic cells in etoposide-treated Annexin V/PI-labeled *HMGB2* KD hESCs relative to etoposide-treated control cells (Figure 4), enhanced caspase-3 and PARP cleavage was detected in the etoposide-treated *HMGB2* KD hESCs by Western blotting (Figure 7A). These data indicate that silencing of either *HMGB1* or *HMGB2* promoted cell death in etoposide-treated hESCs, with the strongest impact observed in *HMGB1* KD cells.

Induction of p53 was already observed in etoposide-untreated *HMGB1* KD hESCs or hNECs (Figure 7), and further enhanced upon treatment of the cells with etoposide for 3 h. However, no significant difference in p53 induction was observed in control cells or cells upon *HMGB* KD treated with etoposide for longer times, i.e., 12 or 24 h (Figure 7). Enhanced cleavage of RIP (a specific marker for necrosis) was consistently observed in etoposide-treated *HMGB1* KD hESCs (Figure 7A). Cleavage of RIP was also observed in untreated and etoposide-treated *HMGB1* KD hNECs and was gradually enhanced upon prolonged etoposide treatment of the cells prepared according to Scenario A, but much less according to Scenario B (Figure 7B,C). In agreement with our previous report [10], untreated *HMGB1* KD hNECs exhibited consistently decreased expression of SOX2 and differentiation marker PAX6 (Figure 7). Expression of SOX2 and PAX6 was further diminished upon prolonged incubation of *HMGB1* and/or *HMGB2* KD hNECs with etoposide but was not observed in the control treated cells (Figure 7B,C). No impact of etoposide and/or *HMGB* KD on the expression of Oct4 was observed in hESCs, demonstrating that the pluripotent state of the cells was preserved (Figure 7A).

### 3.8. Impact of HMGB1/2 KD on Telomere Integrity is Distinct in Etoposide-Treated hESCs and hNECs

Proteins of the shelterin complex associate with telomere ends and help to protect them from triggering DNA repair pathways (telomere “capping”) [47]. Several studies have demonstrated that telomere “uncapping”, e.g., by the exposure of the cells to etoposide [48], brings about a p53-dependent accumulation of DNA damage response factors (such as γ-H2AX and 53BP1) on telomeres, forming Telomere dysfunction-Induced Foci (TIFs) [28,49]. Previously, we have shown by confocal microscopy that deregulation of *HMGB1* and *HMGB2* expression could differentially affect telomere integrity and stability in human ESCs and neuroectodermal cells, as revealed by the detection of TIFs [17].

**Figure 8:**
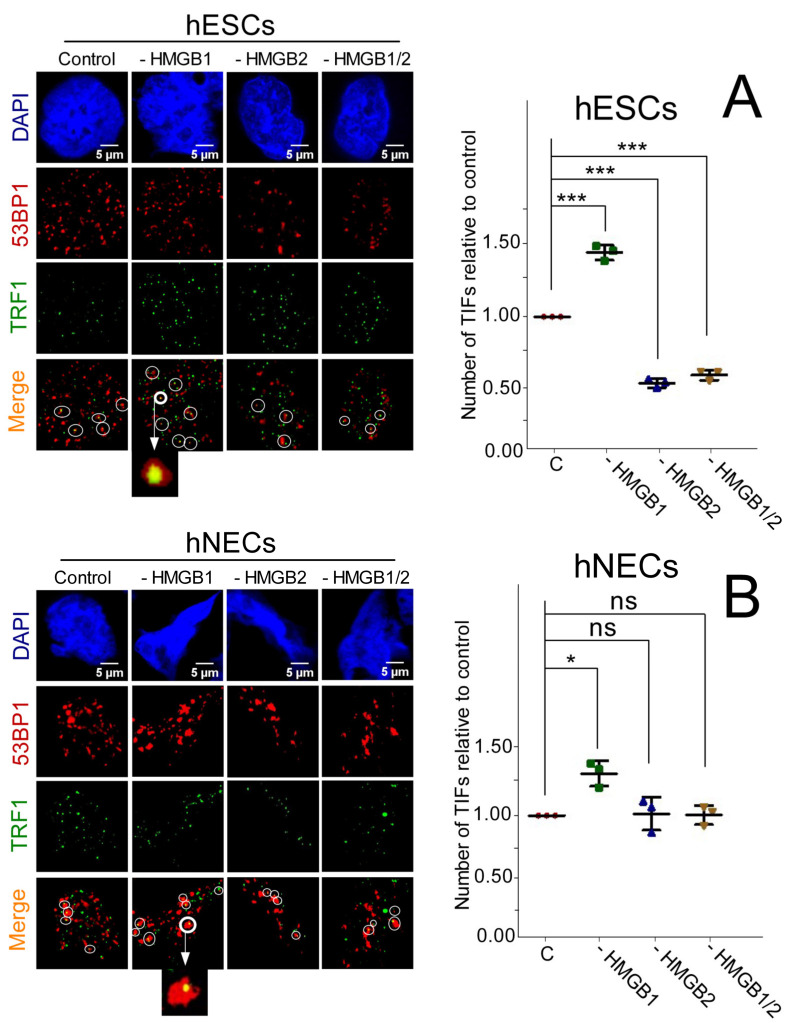
Accumulation of 53BP1 on telomeres in etoposide-treated cells with deregulated expression of *HMGB1*/*2*. (**A**) hESCs or (**B**) hESC-derived neuroectodermal cells (hNECs) prepared according to Scenario A. Left panels: Following etoposide exposure (3.4 μM/3 h), control cells and *HMGB1*- and/or *HMGB2*-depleted cells were processed for immunofluorescence using antibodies against TRF1 (green) and 53BP1 (red) and staining of DNA (blue). Right panels: bar graphs indicate cells with ≥4 colocalization events of 53BP1 with telomeres (TRF1) as number of TIFs (Telomere Dysfunction-Induced Foci) relative to control cells per nucleus. The number of TIFs per nucleus in control cells was arbitrarily set as 1. In each of the cell lines, ~200 nuclei were analyzed using CellProfiler modular image analysis software (https://cellprofiler.org). Colocalization of 53BP1 with telomeres is indicated by circles, and some colocalization events (enlarged) are circled. “C”, cells transfected with empty vector. Statistical differences among samples were evaluated using one-way ANOVA analysis of variance followed by Tukey’s multiple comparison test; *p*-value < 0.05 was considered to indicate significance (*p* > 0.05 = not significant, ns; * *p* < 0.05, and *** *p* < 0.001). Scale bar = 5 μm.

Here we have studied the impact of *HMGB1* and/or *HMGB2* KD in etoposide-treated hESCs or hNECs on telomere integrity and stability by measuring the number of TIFs (co-localization of 53BP1 with TRF1) at telomeres. Colocalization of 53BP1 and TRF1 (a key component of the shelterin complex) was revealed by confocal microscopy and image analysis (see Section 2). An increased number of TIFs per nucleus was detected in etoposide-treated *HMGB1* KD hESCs and hNECs cells relative to control cells (Figure 8). While *HMGB2* or *HMGB1/2* KD in etoposide-treated hESCs resulted in a decreased number of TIFs, no changes in TIFs were recorded under the same conditions in the *HMGB* KD hNECs (Figure 8). Our results demonstrate a distinct impact of HMGB1 and HMGB2 proteins on telomere protection in etoposide-treated hESCs and neuroectodermal cells (Figure 8). The HMGB1 protein promoted telomere stability (as revealed by increased number of TIFs in *HMGB1* KD cells) in both untreated [17] and etoposide-treated hESCs and hNECs (Figure 8). Previously, we have demonstrated that HMGB2 protein can destabilize telomeres (as revealed by decreased number of TIFs in *HMGB2* KD cells) in etoposide-untreated hESCs and hNECs [17]. Interestingly, the destabilization impact of the HMGB2 protein on telomeres was detected only in etoposide-treated hESCs, but not in etoposide-treated neuroectodermal cells (Figure 8).

### 3.9. The Distinct Impact of HMGB2 KD on Telomerase Activity in Etoposide-Treated hESCs and hNECs

Telomerase is critical in the development of cellular immortality and cancer. Telomerase is activated in the majority of human malignancies (reviewed in [50]). Combining telomerase inhibition with apoptosis-inducing drug treatments was shown to increase cell death compared to drug treatment alone ([26,27] and refs. therein). Previously, we demonstrated the distinct impact of *HMGB1* and/or *HMGB2* KD on telomerase activity in mouse and human cells [10,17,28]. We have also demonstrated that HMGB1/2 proteins could enhance expression of the topoisomerase IIα gene [29]. Reports are available demonstrating enhanced telomerase activity in human cancer cells after exposure to the anticancer drug etoposide, a potent topoisomerase IIα inhibitor [23,24].

**Figure 9:**
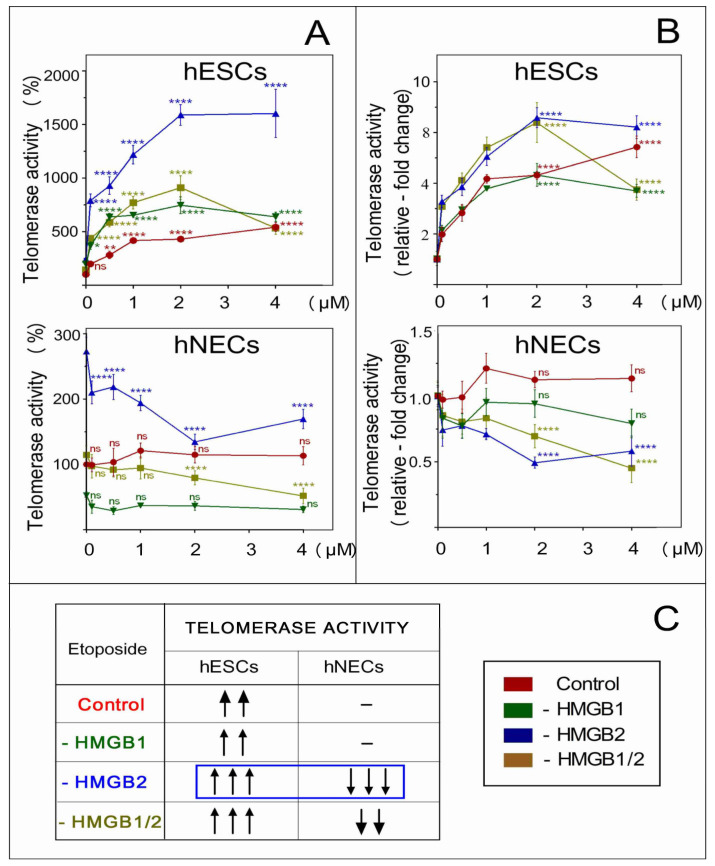
Modulation of telomerase activity by HMGB1 and/or HMGB2 in etoposide-treated hESCs or hNECs. Telomerase activity was determined in hESCs or hESC-derived neuroectodermal cells (hNECs) by qPCR telomeric repeat amplification protocol (TRAP) assay, as detailed in [17]. Cells were treated with etoposide at 0, 0.1, 0.5, 1, 2, or 4 μM for 24 h. (**A**) Telomerase activity relative to etoposide-untreated control cells (set as 100%). (**B**) A recalculation of telomerase activity from (**A**), where both untreated control and *HMGB* KD cells were set as 1. Red line, control cells transfected with empty vector (no *HMGB* KD); green line, *HMGB1* KD; blue line, *HMGB2* KD; dark yellow, *HMGB1* and *HMGB2* KD; ns, not significant. (**C**) A summary of the impact of *HMGB* KD on telomerase activity in etoposide-treated cells. “−”, no change in telomerase activity before and after etoposide treatment; ↑, increased telomerase activity; ↓, decreased telomerase activity (number of arrows indicate the relative strength of impact). Statistical differences among samples were evaluated by one-way ANOVA, followed by Tukey’s multiple comparison test. Values of *p* < 0.05 were considered significant (* *p* < 0.05, ** *p* < 0.01, *** *p* < 0.001, **** *p* < 0.0001).

We have studied the impact of deregulated *HMGB1* and/or *HMGB2* expression on telomerase activity in etoposide-treated hESCs and neuroectodermal cells. We also investigated whether the observed distinct impact of *HMGB* KD on telomere integrity (Figure 8) could be related to changes in telomerase activity in these cells. Control cells or cells upon *HMGB1* and/or *HMGB2* KD were treated with etoposide and the telomerase activity was analyzed by qPCR, as detailed in [17]. We have confirmed that telomerase activity was significantly higher in control (untreated) hESCs than in hESC-derived neuroectodermal cells (hNECs), in agreement with previous reports [10,17]. Several very interesting and unexpected findings were obtained upon the determination of telomerase activity in etoposide-treated hESCs and hNECs. Etoposide treatment of human embryonic stem cells, irrespective of *HMGB* knockdown, resulted in a significant enhancement of telomerase activity. The magnitude of telomerase activity upregulation increased with the etoposide dose and was highest in cells treated with 2‒4 μM for 24 h (Figure 9, top panels A and B). The observed increase in telomerase activity in hESCs not only depended on the concentration of etoposide, but also on the type of *HMGB* KD (Figure 9, top panels A and B). The highest telomerase activity was detected in the etoposide-treated *HMGB2* or *HMGB1/2* KD hESCs. Treatment of control hESCs or *HMGB1* KD hESCs with etoposide resulted in a similar increase in telomerase activity, indicating that HMGB1 was not involved in the etoposide-mediated enhancement of telomerase activity in hESCs (Figure 9B, top panel).

A distinct impact of *HMGB1* or *HMGB2* KD on telomerase activity was observed in etoposide-treated neuroectodermal cells (hNECs). Interestingly, treatment of *HMGB2* or *HMGB1/2* KD hNECs with etoposide markedly decreased telomerase activity in a concentration-dependent manner (Figure 9B, lower panel). Thus, *HMGB2* knockdown in etoposide-treated hESCs resulted in enhanced telomerase activity (indicative of an inhibitory impact of the HMGB2 protein on telomerase activity in the cell), while in the hESC-derived hNECs, loss of *HMGB2* could inhibit telomerase activity (indicative of a stimulatory impact of the HMGB2 protein on the cellular activity of telomerase). Moreover, *HMGB2* KD could enhance telomerase activity in both etoposide-untreated hESCs and hNECs, demonstrating that the HMGB2 protein inhibits the cellular activity of telomerase in the absence of etoposide [10,17]. Telomerase activity was hardly affected, if at all, in the etoposide-treated control or *HMGB1* KD hNECs (Figure 9B, lower panel), suggesting that the HMGB1 protein could not modulate telomerase activity in the etoposide-treated hESCs or hNECs (Figure 9B). The impacts of *HMGB* KD on telomerase activity in etoposide-treated cells are summarized in Figure 9C.

## 4. Discussion

HMGB1 and HMGB2 proteins are currently under investigation due to their involvement in DNA replication, transcription, recombination, repair, inflammation, tumor development, and cell signaling [4,51]. In this report, the main emphasis was on understanding the impact of *HMGB1* and *HMGB2* silencing (knockdown) on stemness of hESCs, cell cycle distribution, apoptosis, and telomerase activity in hESCs and their differentiated derivatives (neuroectodermal cells, hNECs) treated with the anticancer drug etoposide. HMGB1 and HMGB2 proteins are highly related, share >80% identity and differ mainly in the length of their acidic C-tails (reviewed in [4]). Although their DNA binding preferences are similar, their protein binding partners are mostly distinct [4,52]. HMGB1 is indispensable for life, whereas *HMGB2*^−/−^ knockout mice are able to survive with reduced fertility and spermatogenesis defects [34,53,54]. *HMGB1* expression is ubiquitous, but *HMGB2* is widely expressed during embryogenesis [54] and also in adults, mainly in the lymphoid organs and testes [18]. HMGB2 has various functions in distinct differentiation programs, such as erythropoiesis, chondrogenesis, and regulation of neurogenesis ([14,34,55] and refs. therein). The abovementioned differences in the structure, expression, and function of HMGB1 and HMGB2 may provide a basis for understanding their distinct roles in the modulation of apoptosis, differentiation, and telomerase activity, as well as the differences in the efficacy of the anticancer drug etoposide.

*HMGB1* and *HMGB2* expression is augmented in a number of tumors, such as human gastric, breast, and ovarian cancer ([56,57,58] and refs. therein). Metastasis and large tumor size are correlated with high telomerase activity and enhanced HMGB1 and HMGB2 levels, and shown to be associated with lower patient survival (reviewed in [59]). It is possible that HMGB2 may have distinct functions in the context of different cells. In cancer cells ([55,56,57,58,60,61] and refs. therein), the HMGB2 protein could function as a positive regulator of telomerase activity. In noncancer cells (such as mNSCs, neural progenitor cells, mouse embryonic fibroblasts, mESCs, hESCs, or hESC-derived neuroectodermal cells, [10,13,17,62]), it could act as a negative regulator of the cellular activity of telomerase. Etoposide treatment of hNECs reversed the negative impact of HMGB2 on telomerase activity, and the protein could stimulate telomerase activity, as demonstrated by our results. The molecular basis of the different impact of HMGB2 on telomerase activity in control and etoposide-treated cells is unknown, but may be related to distinct mechanisms involving telomerase regulation and/or DNA damage response in hESCs and their differentiated derivatives ([17,63,64] and refs. therein).

Although human embryonic stem cells and cancer cells share many similarities, hESCs do not form tumors following blastocyst implantation, most likely due to the fact that hESCs differentiate when present in an environment that would otherwise drive them to tumorigenesis [65,66]. The stem cell niche (the microenvironment regulating stem cell fate) is characterized by an environment that is nonpermissive with respect to tumor development and possesses the unique ability to reprogram and reverse tumorigenicity. This may also be related to the secretion of soluble factor(s) by hESCs, capable of inhibiting growth of cancer cells, and exposure to such factors might represent a new cancer treatment strategy [65,67]. Whether HMGB proteins are involved in the secretion/production of these factors is unknown.

Although telomerase activity is strongly upregulated in etoposide-treated human pancreatic cancer cells and human leukemia HL-60 cells, it was not observed in other studied cells such as KP-3 and KP-1N [23,24]. A negative correlation was found between increased telomerase activity and the percentage of dead cells after etoposide treatment [23,24], indicating that telomerase upregulation in etoposide-treated cells was likely associated with elements of cellular DNA damage response preceding apoptosis. The latter explanation may also be true for etoposide-treated hESCs [25]. Higher telomerase activity in the etoposide-treated *HMGB2* KD hESCs (relative to control treated cells) may confirm the reported involvement of HMGB proteins in the DNA damage response [6]. Thus, enhanced telomerase activation in etoposide-treated *HMGB2* KD hESCs may reflect augmented DNA damage response in these cells due to the absence of HMGB2 as a DNA damage protective protein.

HMGB1 and HMGB2 not only differently affect the cellular activity of telomerase in etoposide-untreated or -treated hESCs and hNECs [10,17], but the proteins also differentially modulate the sensitivity of cancer cells to distinct anticancer drugs. While *HMGB1* or *HMGB2* KD could enhance the sensitivity of SKOV-3 cells to carboplatin, *HMGB1* KD resulted in a loss of response to the anticancer drug paclitaxel [68]. *HMGB2* KD could also promote sensitivity to another anticancer drug, olaparib [68].

## 5. Conclusions

Our findings on the distinct roles of HMGB1 and HMGB2 in the modulation of telomerase activity in etoposide-treated cells could be of interest for developing strategies for augmenting the efficacy of anticancer drugs [6]. It remains to be established whether the reported distinct impact of *HMGB* KD on the efficacy of anticancer drugs ([6] and refs. therein and in this paper) is correlated with HMGB-dependent changes in the cellular activity of telomerase.

## Supplementary Materials

The following are available online at https://www.mdpi.com/2218-273X/10/10/1450/s1, Figure S1. Etoposide-treated hESCs and hNECs exhibit time- and HMGBs KD-dependent cell-cycle progression and sub-G1 peak formation.
