# Supplementary material for: Nonhistone Proteins HMGB1 and HMGB2 Differentially Modulate the Response of Human Embryonic Stem Cells and the Progenitor Cells to the Anticancer Drug Etoposide
Source: Biomolecules. 2020 Oct 15;10(10):1450. doi: 10.3390/biom10101450 (PMC7602880; doi:10.3390/biom10101450)
Supplement: Supplementary file 1 [file biomolecules-10-01450-s001.pdf]

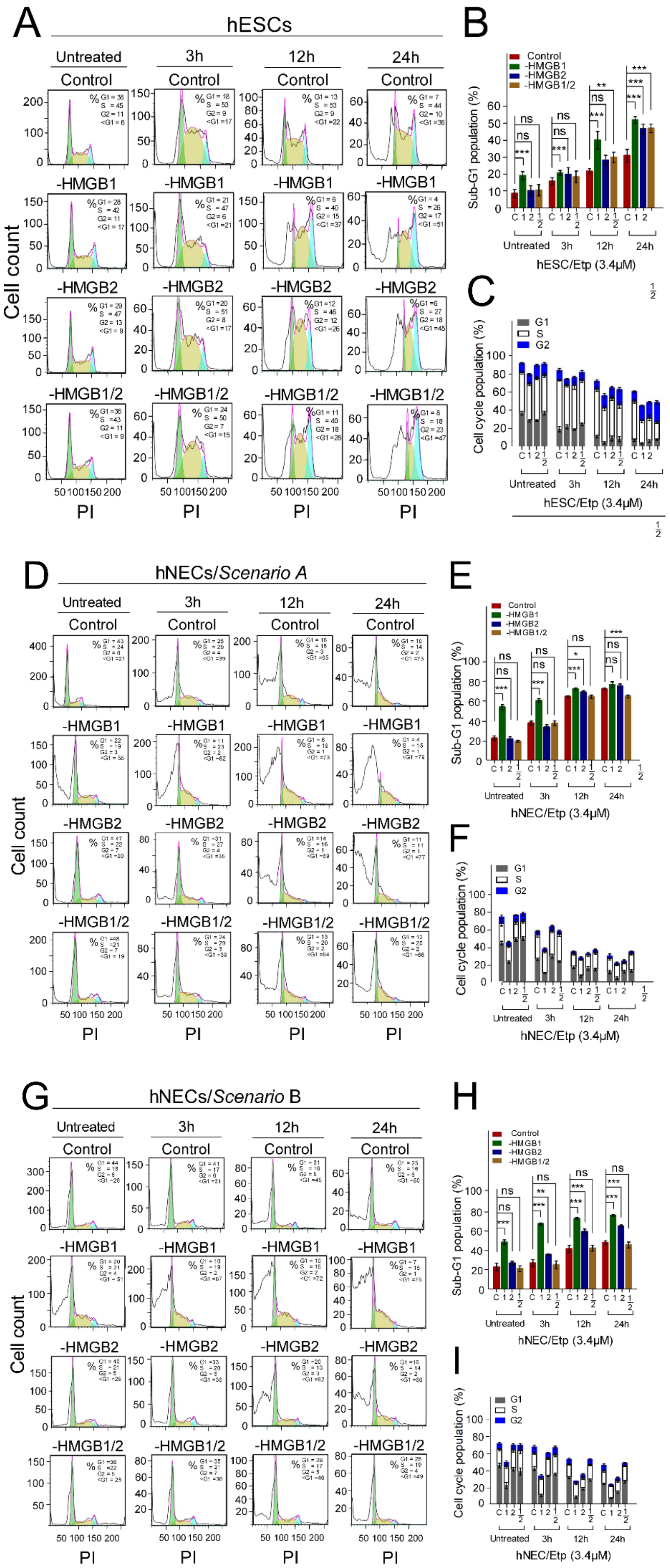
A
hESCs
Untreated
3h
12h
24h
Control
-HMGB1
-HMGB2
-HMGB1/2
Cell count
PI
B
Control
-HMGB1
-HMGB2
-HMGB1/2
Sub-G1 population (%)
Untreated
3h
12h
24h
hESC/Etp (3.4μM)
C
G1
S
G2
Cell cycle population (%)
Untreated
3h
12h
24h
hESC/Etp (3.4μM)
D
hNECs/Scenario A
E
Sub-G1 population (%)
hNEC/Etp (3.4μM)
F
Cell cycle population (%)
hNEC/Etp (3.4μM)
G
hNECs/Scenario B
H
Sub-G1 population (%)
hNEC/Etp (3.4μM)
I
Cell cycle population (%)
hNEC/Etp (3.4μM)

Figure 1. Etoposide-treated hESCs and hNECs exhibit time- and *HMGBs* KD-dependent cell-cycle progression and sub-G1 peak formation. *Panels* A, D and G: Cell cycle profile analysis. Control (empty vector-transfected) cells and cells with lack of HMGB proteins, either untreated or treated with Etop (3.4 μM) for 3-24 h, stained with PI and analyzed by flow cytometry to determine cell cycle distribution of hESCs (*panel* A) and hNECs prepared by either *scenario* A (*panel* D) or *scenario* B (panel G). *Panels* B, E and H: Percentage of sub-G1 fraction in hESCs and hNECs prepared by either *scenario* A or *scenario* B and evaluated by flow cytometry. Representative histograms of percentage of cells in each phase of the cell cycle shown in *panels* A, D and G. Error bars represent SD of three independent experiments. Intensity of propidium iodide stained nuclei, PI; data were analyzed by Bonferroni posttest ($P > 0.05$ not significant, ns; $P < 0.05$ *, $P < 0.01$ **, $P < 0.001$ ***). "C", cells transfected with empty vector; "-HMGB1", cells upon *HMGB1* knockdown; "-HMGB2", cells upon *HMGB2* knockdown; "-HMGB1/2", cells upon *HMGB1* and *HMGB2* knockdown. "1", cells upon *HMGB1* knockdown; "2", cells upon *HMGB2* knockdown; "1/2", cells upon *HMGB1* and *HMGB2* knockdown.
